# AltitudeOmics: Spontaneous Baroreflex Sensitivity During Acclimatization to 5,260 m: A Comparison of Methods

**DOI:** 10.3389/fphys.2019.01505

**Published:** 2019-12-10

**Authors:** Nicolas Bourdillon, Sasan Yazdani, Jean-Marc Vesin, Andrew W. Subudhi, Andrew T. Lovering, Robert C. Roach, Bengt Kayser

**Affiliations:** ^1^Institue of Sport Sciences, University of Lausanne, Lausanne, Switzerland; ^2^Applied Signal Processing Group, Ecole Polytechnique Fédérale de Lausanne, Lausanne, Switzerland; ^3^Altitude Research Center, Department of Medicine, University of Colorado Anschutz Medical Campus, Aurora, CO, United States; ^4^Human Physiology and Nutrition, University of Colorado Colorado Springs, Colorado Springs, CO, United States; ^5^Department of Human Physiology, University of Oregon, Eugene, OR, United States

**Keywords:** baroreflex sensitivity, hypoxia, exercise, altitude, hypercapnia

## Abstract

**Introduction:**

Baroreflex sensitivity (BRS) is essential to ensure rapid adjustment to variations in blood pressure (BP). Spontaneous baroreflex function can be assessed using continuous recordings of blood pressure. The goal of this study was to compare four methods for BRS quantification [the sequence, Bernardi’s (BER), frequency and transfer function methods] to identify the most consistent method across an extreme range of conditions: rest and exercise, in normoxia, hypoxia, hypocapnia, and hypercapnia.

**Methods:**

Using intra-radial artery BP in young healthy participants, BRS was calculated and compared using the four methods in normoxia, acute and chronic hypoxia (terrestrial altitude of 5,260 m) in hypocapnia (hyperventilation), hypercapnia (rebreathing) and during ramp exercise to exhaustion.

**Results:**

The sequence and BER methods for BRS estimation showed good agreement during the resting and exercise protocols, whilst the ultra- and very-low frequency bands of the frequency and transfer function methods were more discrepant. Removing respiratory frequency from the blood pressure traces affected primarily the sequence and BER methods and occasionally the frequency and transfer function methods.

**Discussion/Conclusion:**

The sequence and BER methods contained more respiratory related information than the frequency and transfer function methods, indicating that the former two methods predominantly rely on respiratory effects of BRS. BER method is recommended because it is the easiest to compute and even though it tends to overestimate BRS compared to the sequence method, it is consistent with the other methods, whilst its interquartile range is the smallest.

## Introduction

Blood pressure (BP) must be tightly regulated to guarantee adequate perfusion of organs, especially the brain. BP homeostasis is challenged in numerous situations such as postural change, exercise, and exposure to environmental stresses like heat and altitude. Long and short-term regulation (few heart beats or minutes to lifetime) of BP is achieved by modifications of the vascular tone whilst short-term regulation (few heart beats or minutes) is achieved by heart rate modification (Messerli et al., 2007; Michelini et al., 2015). Altitude induces hypoxemia, leading to vasodilation and BP reduction (Bourdillon et al., 2018). Conversely, hypocapnia induces vasoconstriction and therefore increases BP. Baroreflex is a vital mechanism that triggers variations in heart rate to attenuate variations in BP. The peripheral chemoreceptor-mediated sympathetic excitation in hypoxia results in an increase in baroreflex set point (Halliwill and Minson, 1982) and a decrease in gain (Bernardi et al., 1998; Cooper et al., 2005). During dynamic exercise (walking, running, cycling), cardiac output increases and systemic vascular resistances change (vasodilation in working muscle, vasoconstriction of renal and gastro-intestinal vascular beds) leading to an increased BP, which triggers the baroreflex (Michelini et al., 2015). Yet, mean BP only increases moderately, because there is a resetting of the baroreflex to increased arterial BP as a function of exercise intensity (Bevegård and Shepherd, 1966; Eckberg et al., 1975; Pawelczyk and Raven, 1989; Joyner, 2006) from rest to 75% of maximum oxygen consumption (Potts et al., 1993; Papelier et al., 1994).

Baroreflex sensitivity (BRS) is a measure of baroreflex function. The faster the response to small changes in BP, the more sensitive the autonomic control of BP and the higher the BRS. Originally, BRS quantification gained acceptance by injecting a pressor drug (alpha-adrenergic phenylephrine) which resulted in baroreflex increased vagal efferent activity, lengthening the beat-to-beat intervals, followed by a reduction in sympathetic vasoconstrictor tone within a few seconds delay (La Rovere et al., 2008). There are several components of the baroreflex (cardiac and peripheral). Our focus here is on the cardiac BRS. It is evaluated as the slope of the regression line fitting the sequence of the increases in beat-to-beat intervals with the increases in systolic arterial pressure elicited by the drug. This is called the sequence method, which allows a direct interpretation of the causal link between blood pressure and heart rate changes (Parati et al., 1988).

Spontaneous BRS assessment relies on the same principle but without the injection of a pressor drug. BRS is quantified using any spontaneous consistent sequences of BP vs. beat-to-beat-interval that occur. In the absence of a gold standard (e.g., comparison to the modified Oxford method), we use the sequence method for comparative purposes because it is the most commonly used method for baroreflex analysis. Whilst this method works well in healthy people, the lack of such sequences to fit may be a problem under certain circumstances such as hypoxia or pathological conditions. Therefore, other methods have been developed, known as Bernardi’s ratio of the standard deviations (BER), the frequency method and the transfer function method (Pinna and Maestri, 2002; Bernardi et al., 2010).

The frequency and the transfer function methods both rely on the frequency domain analysis of the blood pressure trace, which is made of the ultra low-, very low-, low- and high- frequency ranges. The low-frequency range has previously been used to describe cardiac BRS during exercise (Fisher et al., 2009) whilst blood pressure fluctuations in the high-frequency range were attributed to respiratory frequency (Frederiks et al., 2000). Interpretation of the ultra- and very low-frequency ranges regarding BRS remains unclear.

Previous studies explored consistency of the various methods (Bernardi et al., 2010) but to the best of our knowledge, there has been no head-to-head comparison of these methods to identify which one is the most consistent across a range of various stressors. Thus, the intent of this study was to compare these four different methods across an extreme range of conditions, rest and exercise in normoxia, hypoxia and hypercapnia during the acclimatization phase to 5,260 m of the AltitudeOmics project (Subudhi et al., 2014), and to formulate recommendations on their use.

## Materials and Methods

The Materials and Methods section has been published previously (Bourdillon et al., 2018), it is reproduced here for readers’ convenience.

### Subject Recruitment and Screening

Twenty-one young, healthy, sea-level residents, average age 21, range 19–23 years, were recruited in the region of Eugene, OR, United States (130 m). Physical examinations and the U.S. Army Physical Fitness Test [APFT, push-ups, sit-ups, and a 3.2-km run (Knapik, 1989)] were performed to characterize health and fitness status. Exclusion criteria included being born at > 1,500 m, having traveled to altitudes > 1,000 m in the past 3 months (including air travel), using prescription medications, smoking, being pregnant or lactating, having a history of serious head injury (loss of consciousness), self or familial history of migraine, known hematologic or cardiovascular abnormality (e.g., sickle cell trait, cardiac arrhythmia), pulmonary function or diffusion capacity for carbon monoxide < 90% of predicted, or failure to meet the minimal age/gender standards for the APFT (Knapik, 1989). BRS results using the sequence method have been published previously (Bourdillon et al., 2018). There is no further redundancy between the present data analysis and other publications from the AltitudeOmics project.

### Ethics Statement

The study was approved by the Institutional Review Boards of the University of Colorado and the University of Oregon and by the Human Research Protection Office of the US Department of Defense and was performed according to the Declaration of Helsinki. The subjects were informed about the procedures and risks and gave written consent prior to participation.

### Experimental Design

Familiarization with the experimental procedures included a graded exercise test up to exhaustion ($\dot{\text{V}}$O_2__peak_ test) to assess the aerobic fitness of the subjects and to ensure that the inclusion criteria were met. After familiarization, the subjects underwent experimental trials near sea level (SL, 130 m; barometric pressure 749 mmHg) and on the 1^st^ (ALT1) and 16^th^ day (ALT16) at 5,260 m (barometric pressure 406 mmHg). For each subject, all ALT measurements were carried out around the same time of day to minimize any confounding effects of the circadian rhythm. During ascent (from 1,525 to 5,260 m) the subjects breathed supplemental oxygen (2 L/min, nasal cannula or mask). Administration of O_2_ was ceased just before ALT1 measurements. This ensured standardized acute exposure at ALT1 and minimized any influence of early acute mountain sickness (AMS) during ALT1. Likewise, no symptoms of AMS were observed at ALT16 because of successful acclimatization. An overview of the entire experimental design of the AltitudeOmics project may be found elsewhere (Subudhi et al., 2014).

### Experimental Protocol

Before entering the experimental room, the subjects laid down in a room dedicated to the insertion of an arterial catheter (20–22 gauge) into a radial artery (Arrow International, Reading, PA, United States) under local anesthesia (2% lidocaine). Arterial blood pressure was measured using this catheter and a calibrated pressure transducer (Deltran^®^, Utah Medical, Midvale, UT, United States) connected to an amplifier (BP amp, ADInstruments, Colorado Springs, CO, United States). After ∼30 min of instrumentation, the subjects underwent the resting protocol, followed by the exercise protocol.

### Resting Protocol

Following 10–15 min of quiet rest in a seated position, each experimental testing session consisted of (1) instrumentation; (2) 10 min in room air for baseline; (3) 10 min with end-tidal partial CO_2_ pressure (PETCO_2_) clamped at 40 mmHg (cl-40); (4) 3 min of voluntary hyperventilation to lower PETCO_2_ to ∼20 mmHg (HVE); and (5) a modified rebreathing test (REB, details below). Stages 3 to 5 of the protocol were carried out in a background of hyperoxia (end-tidal partial O_2_ pressure [PETO_2_] ∼250 mmHg).

### Resting Protocol Experimental Setup

Throughout the protocol, the subjects sat upright and breathed through a mouthpiece attached to a two-way, non-rebreathing valve (Hans-Rudolph 2700, Hans-Rudolph, Shawnee, KS, United States). The breathing circuit allowed switching from room air to either an end-tidal clamping system or a rebreathing system. The end-tidal clamping setup used in the present study was a modified version of the system previously described by Olin et al. (2012). The setup allowed stabilizing PETCO_2_ at 40 mmHg by constantly adding a varying portion of CO_2_ into the inspired gas mixture. Throughout the end-tidal PCO_2_ clamping, we maintained PETO_2_ at ∼250 mmHg by titrating 50% (balanced with N_2_) or 100% O_2_ into the inspiratory reservoir, at SL and ALT, respectively.

### Modified Rebreathing Method

The rebreathing bag was filled with gas to achieve inspired PCO_2_ and PO_2_ of 0 and 300 mmHg, respectively, at each altitude. Subjects were instructed to hyperventilate for 3 min (*part 4*) to lower and then maintain PETCO_2_ at 20 mmHg at both sea level and 5,260 m (in a background PETO_2_ of ∼250 mmHg). Participants were then switched to the rebreathing bag and following two initial deep breaths to mix the gas from the bag with that in the respiratory system, they were instructed to breathe *ad libitum* (*part 5*). The rebreathing tests were terminated when PETCO_2_ reached 50 mmHg, PETO_2_ dropped below 200 mmHg, or the subject reached the end of their hypercapnia tolerance.

### Exercise Protocol

Participants were seated on an electrically braked cycle ergometer (Velotron Elite, Racermate, Seattle, WA, United States). The protocol began with a three-min resting baseline in pedaling position on the ergometer. The subjects then completed four 3-min stages at 70, 100, 130 and 160 Watts, followed by 15 Watts/min increments until they could no longer maintain pedaling > 50 rpm despite strong verbal encouragement. No specific pedaling frequency was required. Maximal power output (Watts) was calculated as: work rate of last stage completed + [(work rate increment) × (time into final stage/duration of stage, in seconds)] (Subudhi et al., 2008). Participants breathed room air throughout the exercise protocol.

### Measurements

#### Data Acquisition

All analog data were sampled and recorded at 200 Hz on a personal computer for off-line analysis (Powerlab 16/30; ADInstruments, Bella Vista, NSW, Australia).

#### Sequence Method

Heart beat-to-beat time intervals were extracted directly from BP recordings. Initially, systolic blood pressure (SBP) peaks were extracted from the BP waveform. Time of systolic peak represented occurrence of heartbeat. However, low sampling rates (<250 Hz) may produce jitter in the estimation of peaks (Merri et al., 1990; Task Force, 1996). For instance, at 200 Hz the highest time resolution is within a confidence interval of 5 ms. To refine the location of heartbeats and the SBP values, a second order polynomial was fitted to each extracted peak using four neighbor samples from the BP waveform (two immediately before and two immediately after). Heartbeats were selected as the location of the maximum of the interpolation polynomial. Furthermore, SBP values were updated as the maximum in their corresponding polynomial. Finally, the inter-beat intervals (IBIs) were created as the interval between successive peaks.

The sequence method is based on the identification of at least three consecutive beats in which a strictly defined increase (or decrease) in SBP is followed by a strictly defined increase (or decrease) in the IBI. Fixed minimal changes were considered for SBP and IBI to validate a sequence. Specifically, a minimum change of 1 mmHg between two consecutive SBP values or of 5 ms for IBI was set as the smallest increase (or decrease) in a sequence (Bernardi et al., 2010). Furthermore, the minimum correlation coefficient between changes in SBP and IBI to validate a sequence was set at 0.85. Finally, a minimum number of five sequences was set to validate a BRS estimate. The sensitivity of the baroreflex is obtained by computing the slope of the regression line between changes in SBP and IBI. All computed slopes are finally averaged to obtain the BRS-Seq. The advantage of this method is that the computations are automatic and standardized, which virtually eliminates intra- and inter-subject measurement variability (La Rovere et al., 2008). The baroreflex nature of these spontaneous beat-to-beat interval-systolic pressure sequences was demonstrated by showing that in cats the number of sequences markedly dropped (−89%) after the surgical opening of the baroreflex loop by sinoaortic denervation (Di Rienzo et al., 2001).

#### Bernardi’s Ratio of the Standard Deviations

In this method, the ratio between the standard deviation of the beat-to-beat intervals and the standard deviation of SBP is calculated (BRS-BER) (Bernardi et al., 2010). All other BRS methods use “selected” variability of the BP trace (i.e., selected relationship between RR intervals and systolic BP or specific frequency bands) whilst BER uses “overall” variability. Yet, the BER method can be used for the determination of BRS. Such a non-specific approach must include non-BRS variability. Heart rate and blood pressure fluctuation are not exclusively determined by the arterial baroreflex. Nevertheless, BER showed good agreement with other standard methods (Maestri et al., 1998; Eckberg, 2009; Bernardi et al., 2010). A possible explanation for this apparent discrepancy between evidence and theory is that in a closed-loop system any cardiovascular change induced by different mechanisms (thus even if not initially originated by the baroreflex) is sensed and influenced by the baroreceptors. Finally, this method is free from the mathematical constraints present in the other methods, and therefore much easier to standardize.

#### Frequency Method

For the spectral method (BRS-F), the IBI and BP time series were regularly resampled at 4 Hz. Then, the square root of the ratio of the autoregressive spectral powers of beat-to-beat intervals and SBP series in the ultra low- (0–0.0033 Hz BRS-FULF) very low- (0.0033–0.05 BRS-FVLF), low- (0.05 Hz–0.15 Hz BRS-FLF), and high- (0.15–0.4 Hz BRS-FHF) frequency range, were calculated, respectively. This computation was performed when coherence between beat-to-beat intervals and SBP was greater than 0.5 in the corresponding passband.

#### Transfer Function Method

In the transfer function method (BRS-TF), resampling of time series was identical to BRS-F and BRS was calculated as the average value of SBP-beat-to-beat cross-spectrum divided by the SBP spectrum in the same frequency ranges as BRS-F.

In all cases, BRS was assessed using a 90-s window immediately before the termination of each resting intervention and exercise stage.

#### Influence of Respiration

Baroreflex sensitivity depends on SBP and IBI fluctuations. However, respiration affects both SBP and IBI via mechanisms that are not necessarily of baroreflex origin. Whether respiratory sinus arrhythmia (RSA) is due to a central mechanism or to the baroreflex mechanism is still debated (Eckberg, 2009; Karemaker, 2009). Previous work attempted to separate the effects of the baroreflex and respiration using metronome-guided respiration and adaptive filtering of the data (Tiinanen et al., 2008), showing that the respiratory rate, but not the pattern (i.e., duration of inspiration and expiration phases) is of primary importance (Paprika et al., 2014). Therefore, to control for a potential effect of hyperventilation on BRS, the respiration frequency was extracted via an autoregressive power spectral density (PSD) estimation of the IBI. The PSD was estimated with an autoregressive order of 50, and the respiration frequency was extracted as that of the largest peak in the range [0.1–0.4] Hz. This IBI frequency band was larger than that used in the conventional heart rate variability (HRV) high frequency band, i.e., [0.15–0.4] Hz, as respiration frequency can migrate to the low frequency band [0.04–0.15] Hz.

### Statistical Analysis

**FIGURE 1:**
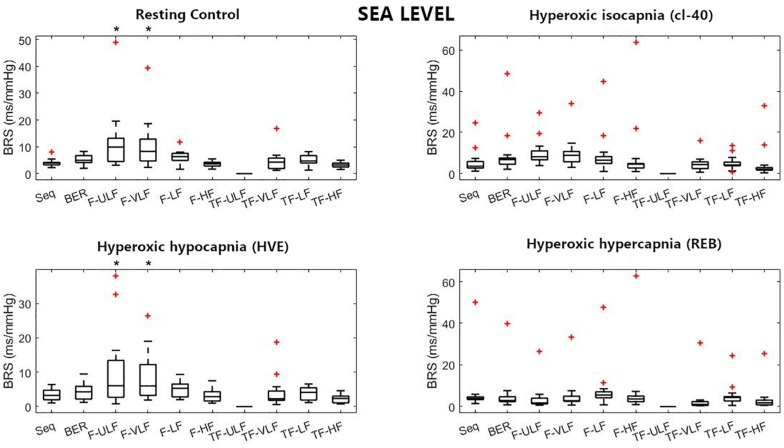
Baroreflex sensitivity (BRS) estimation for the resting protocol at sea level. cl-40, clamp 40 mmHg of inspired CO_2_; HVE, hyperventilation; REB, rebreathing up to PACO_2_ of 50 mmHg. +Denotes outliers. ^∗^Different from Seq (*p* < 0.05).

**FIGURE 2:**
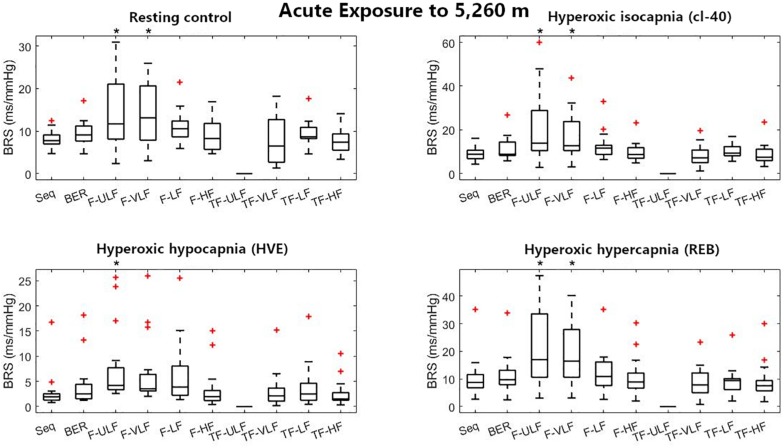
Baroreflex sensitivity estimation for the resting protocol on the first day at 5,260 m. cl-40, clamp 40 mmHg of inspired CO_2_; HVE, hyperventilation; REB, rebreathing up to PACO_2_ of 50 mmHg. +Denotes outliers. ^∗^Different from Seq (*p* < 0.05).

**FIGURE 3:**
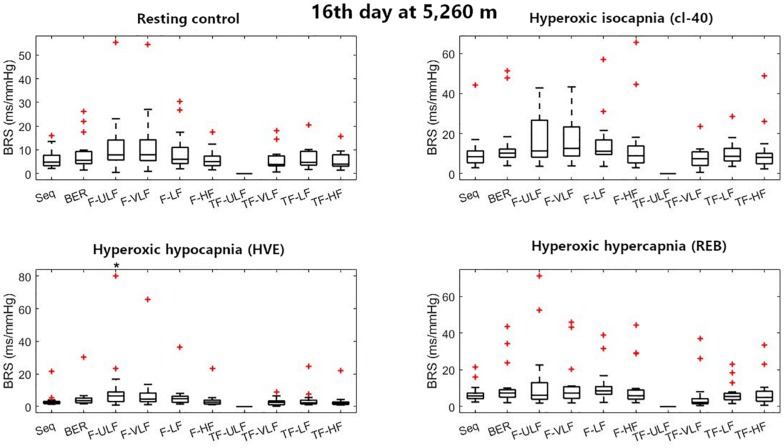
Baroreflex sensitivity estimation for the resting protocol on the 16^th^ day at 5,260 m. cl-40, clamp 40 mmHg of inspired CO_2_; HVE, hyperventilation; REB, rebreathing up to PACO_2_ of 50 mmHg. +Denotes outliers. ^∗^Different from Seq (*p* < 0.05).

Figures 1–3 display Tukey boxplots of the data in which the horizontal line inside the boxes denotes the median, whilst the upper and lower lines of the boxes represent the 75^th^ and 25^th^ percentiles, respectively. The upper and lower whiskers denote the highest and lowest data points within the 1.5 inter quartile range (Frigge et al., 1989). This corresponds to approximately ± 2.7σ and 99.3% coverage of the data (McGill et al., 1978). Outliers were marked using the + sign. Smaller inter quartile range on the figures denote good consistency across the participants when making recommendations on which method should be used. Bias, reproducibility coefficient and coefficient of variations are available in the Supplementary Material.

Two-way ANOVAs were performed to compare the various methods in each condition in the rest protocol, and each exercise intensity (PRE, 70W, 100W, 130W, 160W, and MAX) in the exercise protocol across SL, ALT1, and ALT16. The Tukey-Kramer *post hoc* test was performed when appropriate. The alpha level for significance was set at 0.05 and is reported rounded to three digits after the decimal point. All analyses were completed using MATLAB^®^ (MathWorks, Natick, MA, United States). The Bland & Altman parameters are reported in Supplementary Tables S1, S2 for the rest protocol and Supplementary Table S3 for the exercise protocol. *p*-Values for the Bland & Altman are reported in these tables and may differ from the ANOVA significant levels indicated on the figures. In our case, ANOVAs are more reliable as they take into account the two ways (time × altitude) and that the same participants were measured in each condition. The Bland & Altman method is used to analyze the agreement between two different assays by determining the agreement between two methods used to measure the same parameter. The graphical analysis plots the difference between the two methods versus the mean (or median) of the two methods (Bland and Altman, 1986). In the present work, the median is used to make the analyses more robust between conditions and less sensitive to the extreme values of some of the participants.

## Results

Twenty one participants were included in this study (12M/9F) aged 20.8 ± 1.4 years, height 175.8 ± 7.9 cm, weight 69.7 ± 9.0 kg, and BMI 22.4 ± 1.8 kg/m^2^.

Variations of BRS along the various experimental conditions and their physiological correlates are detailed in a previous paper (Bourdillon et al., 2018). This report focuses on the comparison between the four methods used to assess BRS. Briefly, resting heart rate increased in hypoxic conditions (77 ± 17; 90 ± 12, *p* < 0.05; 97 ± 15, *p* < 0.05, bpm SL, ALT1, and ALT16, respectively, all *p*-values vs. SL) whilst heart rate at maximal exercise decreased (185 ± 14; 170 ± 12, *p* < 0.05; 166 ± 13, *p* < 0.05, bpm, SL, ALT1, and ALT16, respectively, all *p*-values vs. SL). Mean resting BP was unchanged in hypoxic conditions (95 ± 11; 94 ± 9; 92 ± 9 mmHg, SL, ALT1, and ALT16, respectively), whilst it decreased at maximal exercise (121 ± 8; 109 ± 10, *p* < 0.05; 110 ± 11, *p* < 0.05, mmHg, SL, ALT1, and ALT16, respectively, all *p*-values vs. SL).

### Resting Protocol Results

Figure 1 shows BRS estimation during the rest protocol at SL. When compared with the Seq method, BRS was significantly higher when estimated using F-ULF and F-VLF during rest and HVE. TF-ULF estimate was arbitrarily set at 0 because not relevant (actual values were close to zero).

Figure 2 shows BRS estimation during the rest protocol at ALT1. When compared with the Seq method, BRS was significantly higher when estimated using F-ULF and F-VLF in almost all conditions.

Figure 3 shows BRS estimation during the rest protocol at ALT16. When compared with the Seq method, BRS was consistent across all methods in all conditions except F-ULF during HVE.

Bland & Altman parameters for the resting protocol are detailed in Supplementary Tables S1, S2. In the latter, respiration frequency has been removed from the BP signal.

### Exercise Protocol Results

**FIGURE 4:**
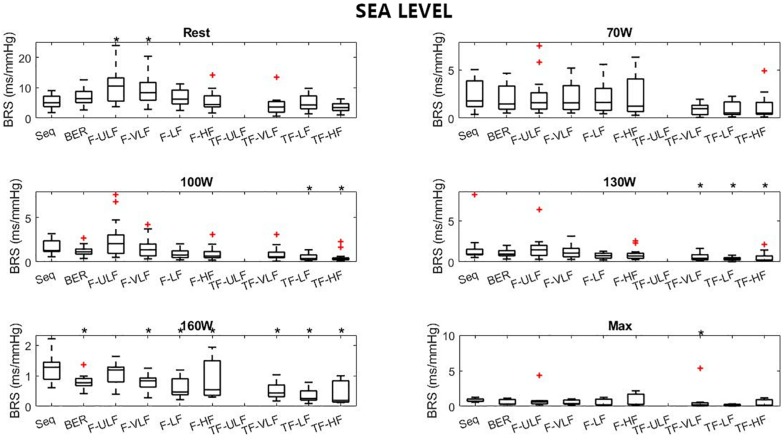
Baroreflex sensitivity estimation for the exercise protocol at sea level. +Denotes outliers. ^∗^Different from Seq (*p* < 0.05).

Figure 4 shows BRS estimation during the exercise protocol at SL. When compared with the Seq method, BRS was significantly lower when estimated using the TF method from 100 W up. All methods underestimated BRS at 160 W compared to Seq.

**FIGURE 5:**
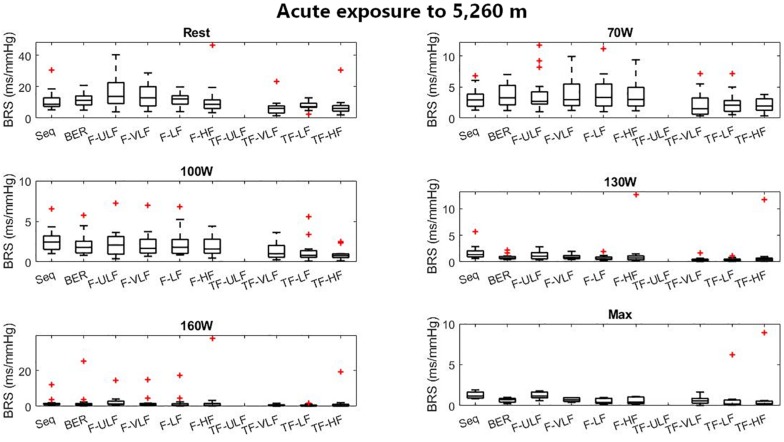
Baroreflex sensitivity estimation for the exercise protocol on the first day at 5,260 m. +Denotes outliers.

Figure 5 shows BRS estimation during the exercise protocol at ALT1. No differences between methods were observed.

**FIGURE 6:**
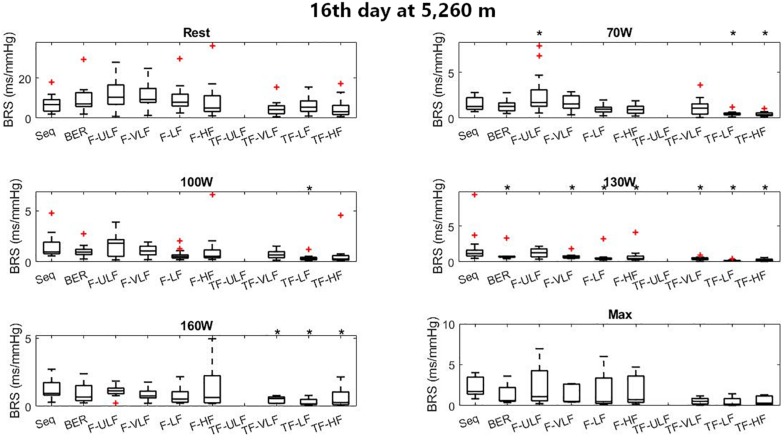
Baroreflex sensitivity estimation for the exercise protocol on the 16^th^ day at 5,260 m. +Denotes outliers. ^∗^Different from Seq (*p* < 0.05).

Figure 6 shows BRS estimation during the exercise protocol at ALT16. Somewhat comparable to SL, BRS was significantly lower when estimated using the TF method from 70 W up. All methods underestimated BRS at 130 W compared to Seq.

### Influence of Respiration on BRS

Supplementary Figures S1–S6 report the same data as Figures 1–6 respectively but with respiration removed. Tables 1, 2 summarize when removing respiration had a significant effect on BRS estimation during rest and exercise.

**TABLE 1:** Effect of removing respiration on BRS estimation during the rest protocol.

		**Seq**	**BER**	**F-ULF**	**F-VLF**	**F-LF**	**F-HF**	**TF-VLF**	**TF-LF**	**TF-HF**
SL	Rest	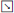 < 0.01	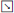 < 0.05	≈	≈	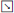 < 0.05	≈	≈	≈	≈
	HVE	≈	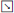 < 0.05	≈	≈	≈	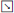 < 0.01	≈	≈	≈
	cl-40	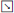 < 0.01	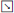 < 0.01	≈	≈	≈	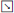 < 0.01	≈	≈	≈
	REB	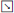 < 0.01	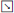 < 0.05	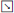 < 0.05	≈	≈	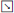 < 0.05	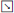 < 0.05	≈	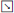 < 0.05
ALT1	Rest	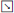 < 0.01	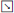 < 0.01	≈	≈	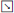 < 0.01	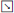 < 0.05	≈	≈	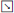 < 0.01
	HVE	≈	≈	≈	≈	≈	≈	≈	≈	≈
	cl-40	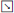 < 0.05	≈	≈	≈	≈	≈	≈	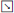 < 0.05	≈
	REB	≈	≈	≈	≈	≈	≈	≈	≈	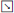 < 0.05
ALT16	Rest	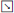 < 0.05	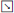 < 0.05	≈	≈	≈	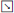 < 0.05	≈	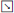 < 0.01	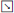 < 0.05
	HVE	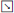 < 0.01	≈	≈	≈	≈	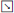 < 0.05	≈	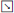 < 0.05	≈
	cl-40	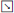 < 0.05	≈	≈	≈	≈	≈	≈	≈	≈
	REB	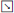 < 0.05	≈	≈	≈	≈	≈	≈	≈	≈

*Arrows indicate increase or decrease. *p*-Values are given with two decimal precision against the same condition with respiration. ≈Denotes non-significant difference between BRS estimated with and without respiration.*

**TABLE 2:** Effect of removing respiration on BRS estimation during the exercise protocol.

		**Seq**	**BER**	**F-ULF**	**F-VLF**	**F-LF**	**F-HF**	**TF-VLF**	**TF-LF**	**TF-HF**
SL	Rest	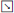 < 0.01	≈	≈	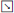 < 0.05	≈	≈	≈	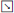 < 0.05	≈
	70W	≈	≈	≈	≈	≈	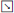 < 0.05	≈	≈	≈
	100W	≈	≈	≈	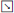 < 0.05	≈	≈	≈	≈	≈
	130W	≈	≈	≈	≈	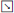 < 0.01	≈	≈	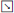 < 0.01	≈
	160W	≈	≈	≈	≈	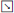 < 0.05	≈	≈	≈	≈
	Max	≈	≈	≈	≈	≈	≈	≈	≈	≈
ALT1	Rest	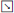 < 0.01	≈	≈	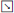 < 0.05	≈	≈	≈	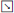 < 0.05	≈
	70W	≈	≈	≈	≈	≈	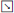 < 0.05	≈	≈	≈
	100W	≈	≈	≈	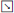 < 0.05	≈	≈	≈	≈	≈
	130W	≈	≈	≈	≈	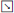 < 0.01	≈	≈	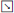 < 0.01	≈
	160W	≈	≈	≈	≈	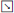 < 0.05	≈	≈	≈	≈
	Max	≈	≈	≈	≈	≈	≈	≈	≈	≈
ALT16	Rest	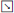 < 0.01	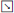 < 0.05	≈	≈	≈	≈	≈	≈	≈
	70W	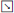 < 0.05	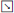 < 0.05	≈	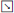 < 0.05	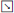 < 0.05	≈	≈	≈	≈
	100W	≈	≈	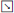 < 0.05	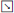 < 0.05	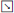 < 0.05	≈	≈	≈	≈
	130W	≈	≈	≈	≈	≈	≈	≈	≈	≈
	160W	≈	≈	≈	≈	≈	≈	≈	≈	≈
	Max	≈	≈	≈	≈	≈	≈	≈	≈	≈

*Arrows indicate increase or decrease. *p*-Values are given with two decimal precision against the same condition with respiration. ≈Denotes non-significant difference between BRS estimated with and without respiration.*

Table 3 shows which method is recommended depending on the stressor used.

**TABLE 3:** Recommendations of methods depending on the stressor used.

		**Seq**	**BER**	**F**	**TF**
Respiration	Rest	OK	OK	LF-HF OK	OK
	Acute hypoxia	OK	OK	LF-HF OK	OK
	Chronic hypoxia	OK	OK	OK	OK
	Hypocapnia	OK	OK	LF-HF OK	OK
	Hypercapnia	OK	OK	OK	OK
	Light exercise	OK	OK	OK	OK
	Moderate exercise	OK	OK	OK	Underestimated
	Severe exercise	OK	OK	OK	OK
Respiration removed	Rest	Underestimated	Overestimated	Overestimated	OK
	Acute hypoxia	Underestimated	Overestimated	Overestimated	OK
	Chronic hypoxia	Underestimated	Overestimated	Overestimated	OK
	Hypocapnia	OK	OK	OK	OK
	Hypercapnia	Underestimated	Overestimated	Overestimated	OK
	Light exercise	OK	OK	OK	OK
	Moderate exercise	Not recommended	OK	OK	OK
	Severe exercise	Not recommended	Not recommended	Not recommended	Not recommended

*Overestimate and underestimate labels are based on the significant differences (ANOVA, *p* < 0.05) indicated in Figures 1–6 and Supplementary Material.*

Bland & Altman parameters for the exercise protocol are detailed in Supplementary Table S3. Removing respiration during exercise resulted in doubtful comparison between methods and therefore is not reported (also see below). During exercise, baroreflex activity decreased drastically as previously reported on the same set of participants (Bourdillon et al., 2018) whilst RSA increased. The beat-to-beat-interval time series variability became dependent only on RSA as exercise intensity increased. Therefore, removing respiration frequency led to a flat beat-to-beat interval shape, which made it hard to identify sequences for BRS estimation using the Seq method.

## Discussion

This study compared four BRS estimation methods across an extreme range of conditions including rest and exercise, in hypoxia, hypocapnia, and hypercapnia in order to make recommendations on which method is most consistent depending on the stressor used. The main findings were: (1) The sequence and BER methods showed good agreement throughout whilst the frequency and transfer function methods were more discrepant (see Supplementary Material for detailed Bland & Altman parameters^1^); (2) conversely, when removing respiration from the blood pressure signal, the sequence and BER methods were more affected than the frequency and transfer function methods.

When it comes to choosing a method, BER seems the best candidate because (1) it is the easiest to compute; (2) it is consistent with the other methods, even though it tends to overestimate with respect to the sequence method; and (3) its interquartile range is smaller compared to that of the other methods, indicating it is more consistent across participants. Also, this method is free from the mathematical constraints present in the other methods, and therefore much easier to standardize (Bernardi et al., 2010).

### BRS Estimation at Rest

Previous comparative studies showed fair agreement between BRS estimation methods in homogeneous groups of humans (Maestri et al., 1998; Pitzalis et al., 1998; Colombo et al., 1999; Laude et al., 2004). Comparing various populations, high-pass filtering further improved the agreement between methods (Bernardi et al., 2010). In the present study, a homogeneous population was exposed to very challenging situations. There was good agreement between Seq, BER and TF methods and not as good with the frequency method. Overestimation of BRS was quasi-systematic using the ULF and VLF bands. These bands represent long-term variations in the baroreflex function and may be associated to progressive changes in catecholamines and slow drifts in the blood pressure signal. Decreased plasma and increased urinary excretion of catecholamines have been reported during acute hypoxia (for review, Rostrup, 1998), whilst in chronic hypoxia, plasma catecholamine levels may normalize. In our experimental setup changes in plasma catecholamine levels were likely a confounding factor for BRS estimation in those frequency bands. At ALT1 and ALT16, during REB, high doses of CO_2_ resulted in very strong ventilatory drive (Bourdillon et al., 2018), which potentially masked the plasma catecholamine variations, hence the absence of significant differences compared to Seq.

### BRS Estimation During Exercise

Baroreflex sensitivity estimation during exercise was consistent between methods as the decrease in BRS was detectable in all methods when exercise intensity increased and hypoxia exposure shifted from ALT1 to ALT16 (Figures 4–6). However, compared to the other methods TF underestimated BRS at almost all exercise stages. BRS estimates become very small when exercise intensity increases, which may make the Seq, F, and TF methods unstable. The numerous constraints for these methods were established for a resting sea level condition. They make Seq look at periods of parallel changes only, whereas F and TF methods look at fluctuations at specific frequencies only. There is no evidence that the established constraints do not induce a bias when estimating BRS under such a challenging situation as incremental exercise performed until exhaustion at high altitude.

### Influence of the Respiratory Rate on BRS Estimation

One of the main physiological factors affecting BRS is the respiratory rate (Horsman et al., 2015). Removing respiration lowered primarily Seq, then BER and occasionally F-LF, F-HF, TF-LF and TF-HF, during the resting protocol. Therefore, Seq and BER contained information on the influence of respiration on blood pressure, whilst the other methods were less sensitive. It remains unclear whether blood pressure and beat-to-beat interval fluctuations coinciding with respiration are of baroreflex origin or arise from another central neural mechanism (Diaz and Taylor, 2006; Tan and Taylor, 2011). Without controlled respiratory rate (with a metronome for example) disentangling the effects of respiratory rate on BRS from the effects of blood pressure oscillation frequency is very difficult if not impossible (Goldstein et al., 1982; Pagani et al., 1988; La Rovere et al., 2008; Bourdillon et al., 2018). However, lowered BRS estimation when removing respiration in Seq and BER indicate that those methods report for the major part the effects of breathing on BRS and for a minor part the effect of blood pressure oscillation frequency. The frequency methods were more balanced and reported in roughly equivalent proportions the two phenomena.

During progressive exercise to exhaustion, there is a resetting of baroreflex to increased arterial pressures (Bevegård and Shepherd, 1966; Eckberg et al., 1975; Pawelczyk and Raven, 1989; Joyner, 2006) which proportionally make the effects of baroreflex on beat-to-beat intervals smaller and the effects of respiratory sinus arrhythmia larger. Also, as beat-to-beat interval decreases drastically, the possibility of variation with regards to blood pressure is much less if not absent. Therefore, the estimate of BRS using Seq is drastically decreased and even more when respiration is removed. The other methods seem to better ‘adapt’ to the exercise condition. The ULF and VLF bands were generally more affected by respiration removal during exercise than during rest. There may be a further confounding factor as during exercise there are important increases in plasma and neural catecholamine levels, associated to increased arterial tone and vasodilation in working muscles, which may flatten the long-term variations in blood pressure.

### Limitations

Guideline recommendations are a window duration between 2 and 5 min for frequency analyses (Task Force, 1996). We are slightly under the lower limit, but as stated above this was imposed by the extreme experimental conditions. This may have made the frequency analyses a little less stable and added noise in our dataset.

The effects of respiration on BRS were only estimated from a frequency point of view. However, tidal volume may also interfere with BRS (Taha et al., 1995; St Croix et al., 1999). Greater tidal volumes (typically hypercapnia and high exercise intensities) may increase the influence of respiration on BRS.

Removing respiration lowers BRS estimate. During exercise, BRS seems to reach its lower limit, and removing respiration during exercise resulted in barely significant BRS estimate for most methods and Bland & Altman computations. Therefore, no table reports Bland & Altman parameters for methods comparison with respiration removed during exercise.

Baroreflex sensitivity in premenopausal females varies across the menstrual cycle. Given the design of this field study, it would have been impractical to control for menstrual cycle across the period of acclimatization. Variations due the menstrual cycle may have added noise in the recordings of the 9 females included in this study.

Using the sequence method, positive and negative sequences were isolated and treated separately, which did not significantly change the results compared to pooling. When more than three consecutive points constituting a sequence were found BRS was calculated with and without overlap of those points, which again did not significantly change the results. The sequence method has a number of limits concerning the criteria about what sequences can be used, but we have taken as many precautions as possible to ensure that the reported BRS represent as accurately as possible what is actually ongoing during acclimatization to altitude. Also, a 90-s window for the sequence method is short, usually 5-min window are used (Ogoh et al., 2005) but, in the context of AltitudeOmics 90 s was the longest steady state that could be tolerated by the participants in hypercapnic condition and at several exercise stages, especially in hypoxic conditions. Although this may limit the number of sequences detected and would influence the overall gain, we have the lower limit of 5 sequences to validate a BRS measurement, as usual in studies using the sequence method. A strength of our study is the use of intra-arterial catheterization for the continuous measurement of blood pressure. Therefore, the signal was clean (low noise) and better delineated than in the great majority of other studies, where generally finger cuff coupled to photo-plethysmography originated signals were recorded. Therefore, the technique presently used (interpolation coupled to maximum finding) is deemed of good quality.

This study is part of the AltitudeOmics project which primary goal was not to explore BRS function. Therefore, there was no condition with controlled respiratory frequency using a metronome, which would have allowed better determination of respiratory and blood pressure oscillations on BRS estimation. Within this article all methods are compared to the sequence method, but there was no comparison to a gold standard method such as the modified Oxford method, therefore methods are only compared between them and under or over-estimation of BRS is only considered as such. However, the AltitudeOmics database richness and the quality of the raw intra-arterial (BP) data deserved being reported to the scientific community.

## Conclusion

This study is the first to report systematic comparison of four BRS estimation methods using blood pressure data obtained with an arterial catheter, during acute, and chronic exposure to 5,260 m, at rest and during dynamic exercise to exhaustion. The sequence and BER methods for BRS estimation showed good coherence but contained more respiratory related information than the frequency and transfer function methods, indicating that the first two methods indicate for a major part respiratory effect on BRS. BER method is recommended because it is the easiest to compute, it is consistent with other methods, even though it tends to overestimate compared to the sequence method, whilst its interquartile range is the smallest, meaning it is more consistent across participants.

## Author’s Note

This paper is part of a series titled “AltitudeOmics” that together represent a group of studies that explore the basic mechanisms controlling human acclimatization to hypoxia and its subsequent retention. Many people and organizations have invested enormous amounts of time and resources to make AltitudeOmics a success.

## Data Availability Statement

All datasets generated for this study are included in the article/Supplementary Material.

## Ethics Statement

This study was approved by the Institutional Review Boards of the University of Colorado and the University of Oregon and by the Human Research Protection Office of the US Department of Defense and was performed according to the Declaration of Helsinki. The patients/participants provided their written informed consent to participate in this study.

## Author Contributions

AL, AS, and RR conceived and designed the experiments, and managed the ethics process. All authors listed in Subudhi et al. (2014) performed the experiments. NB, SY, and J-MV analyzed the data. NB interpreted the data, wrote the first version of the manuscript, and prepared the figures. SY, AS, AL, RR, JM-V, and BK revised the manuscript. All authors approved the final version of the manuscript.

## Conflict of Interest

The authors declare that the research was conducted in the absence of any commercial or financial relationships that could be construed as a potential conflict of interest.

## Supplementary Material

FIGURE S1 Baroreflex sensitivity BRS estimation for the resting protocol at sea level, respiration removed. cl-40, clamp 40 mmHg of inspired CO_2_; HVE, hyperventilation; REB, rebreathing up to PACO_2_ of 50 mmHg. +Denotes outliers. ^∗^Different from Seq (*p* < 0.05).

FIGURE S2 Baroreflex sensitivity estimation for the resting protocol on the first day at 5,260 m, respiration removed. cl-40, clamp 40 mmHg of inspired CO_2__;_ HVE, hyperventilation; REB, rebreathing up to PACO_2_ of 50 mmHg. +Denotes outliers. ^∗^Different from Seq (*p* < 0.05).

FIGURE S3 Baroreflex sensitivity estimation for the resting protocol on the 16^th^ day at 5,260 m, respiration removed. cl-40, clamp 40 mmHg of inspired CO_2__;_ HVE, hyperventilation; REB, rebreathing up to PACO_2_ of 50 mmHg. +Denotes outliers. ^∗^Different from Seq (*p* < 0.05).

FIGURE S4 Baroreflex sensitivity estimation for the exercise protocol at sea level, respiration removed. +Denotes outliers. ^∗^Different from Seq (*p* < 0.05).

FIGURE S5 Baroreflex sensitivity estimation for the exercise protocol on the first day at 5,260 m, respiration removed. +Denotes outliers. ^∗^Different from Seq (*p* < 0.05).

FIGURE S6 Baroreflex sensitivity estimation for the exercise protocol on the 16^th^ day at 5,260 m, respiration removed. +Denotes outliers. ^∗^Different from Seq (*p* < 0.05).

TABLE S1 Bland & Altman parameters during the rest protocol.

TABLE S2 Bland & Altman parameters during the rest protocol, without respiration.

TABLE S3 Bland & Altman parameters during the exercise protocol.
