# Supplementary figures and images for: AltitudeOmics: Spontaneous Baroreflex Sensitivity During Acclimatization to 5,260 m: A Comparison of Methods
Source: Front Physiol. 2019 Dec 10;10:1505. doi: 10.3389/fphys.2019.01505 (PMC6914841; doi:10.3389/fphys.2019.01505)

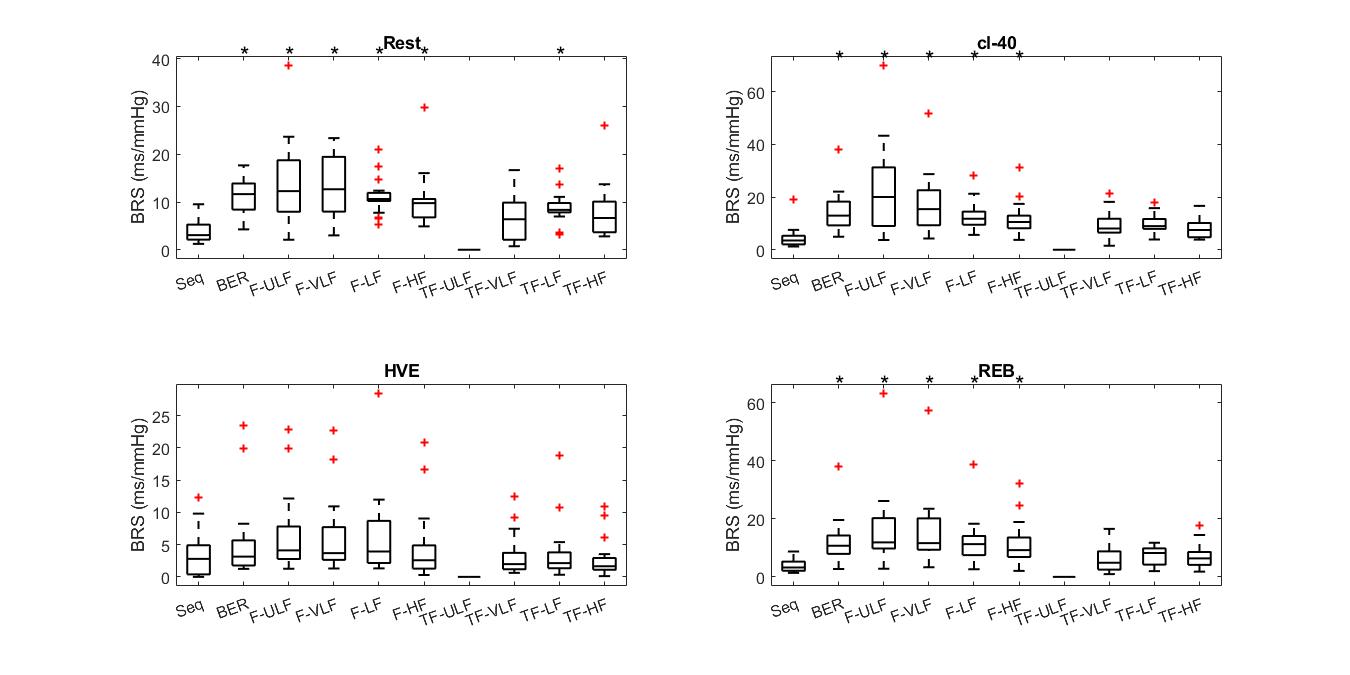

Supplement: FIGURE S1 — Baroreflex sensitivity BRS estimation for the resting protocol at sea level, respiration removed. cl-40, clamp 40 mmHg of inspired CO2; HVE, hyperventilation; REB, rebreathing up to PACO2 of 50 mmHg. +Denotes outliers. ∗Different from Seq (p < 0.05). [file Image_1.JPEG]

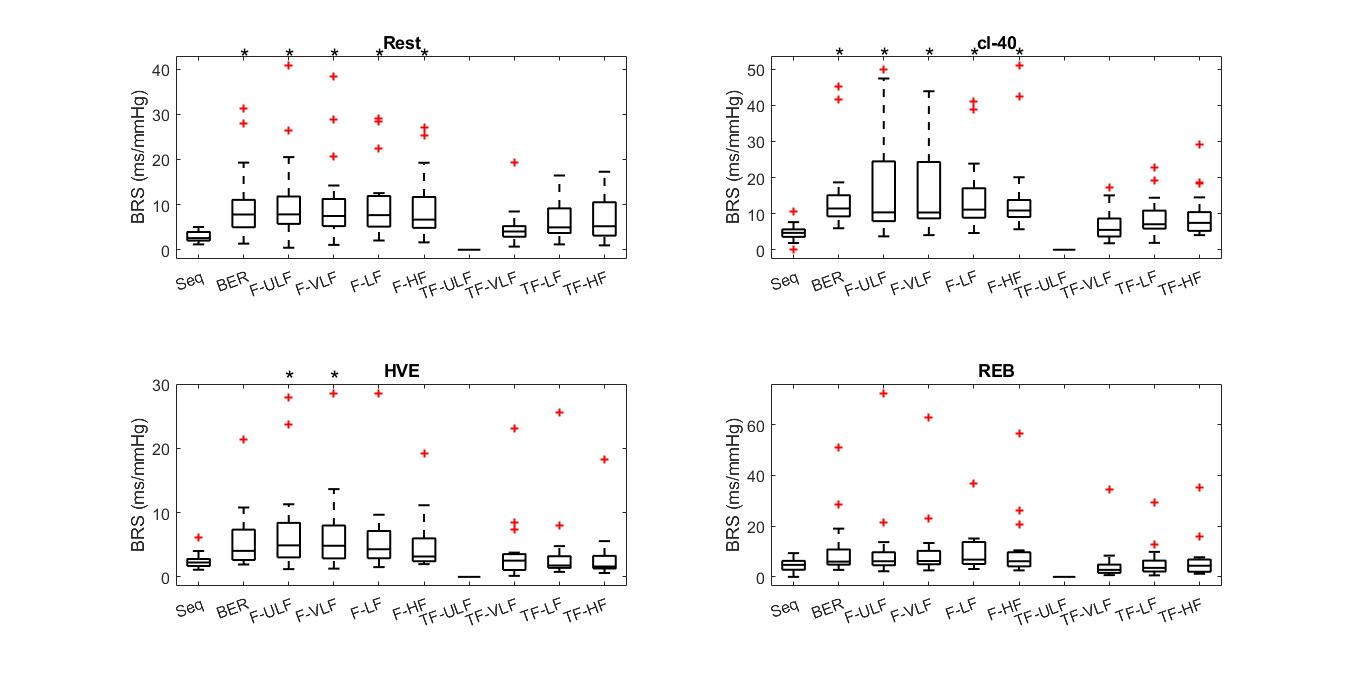

Supplement: FIGURE S2 — Baroreflex sensitivity estimation for the resting protocol on the first day at 5,260 m, respiration removed. cl-40, clamp 40 mmHg of inspired CO2; HVE, hyperventilation; REB, rebreathing up to PACO2 of 50 mmHg. +Denotes outliers. ∗Different from Seq (p < 0.05). [file Image_2.JPEG]

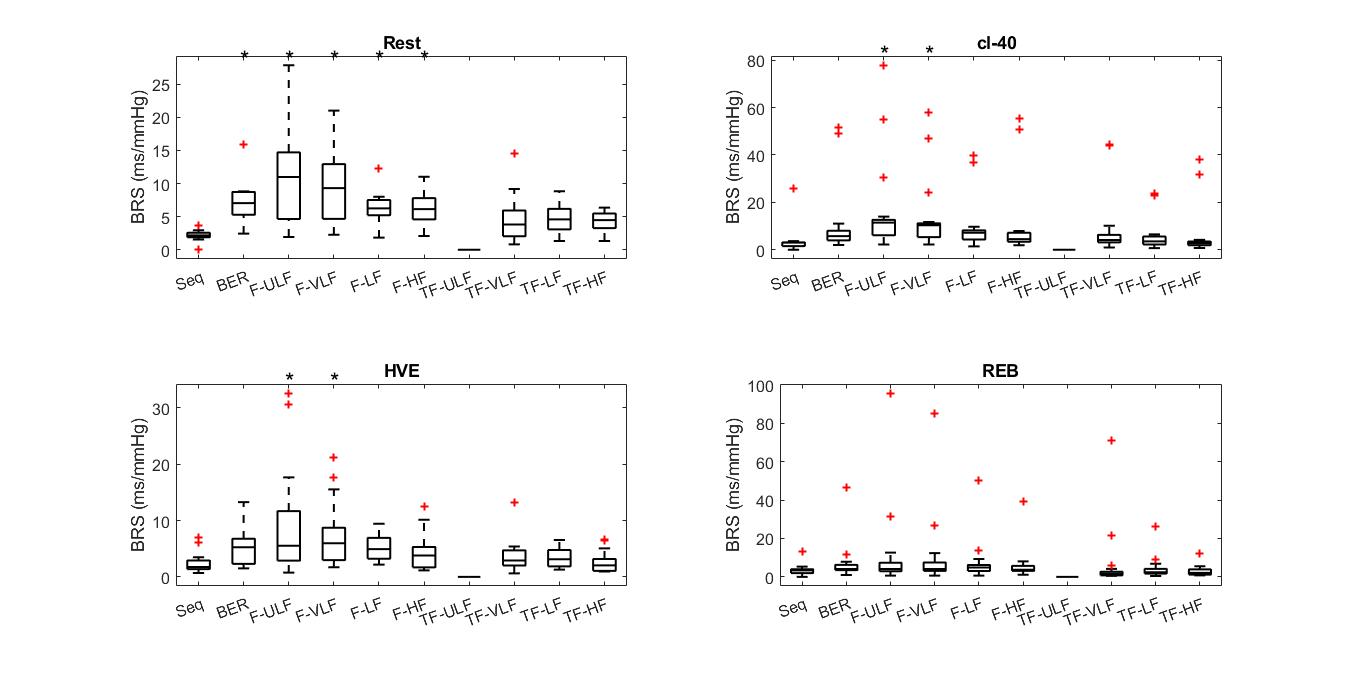

Supplement: FIGURE S3 — Baroreflex sensitivity estimation for the resting protocol on the 16th day at 5,260 m, respiration removed. cl-40, clamp 40 mmHg of inspired CO2; HVE, hyperventilation; REB, rebreathing up to PACO2 of 50 mmHg. +Denotes outliers. ∗Different from Seq (p < 0.05). [file Image_3.JPEG]

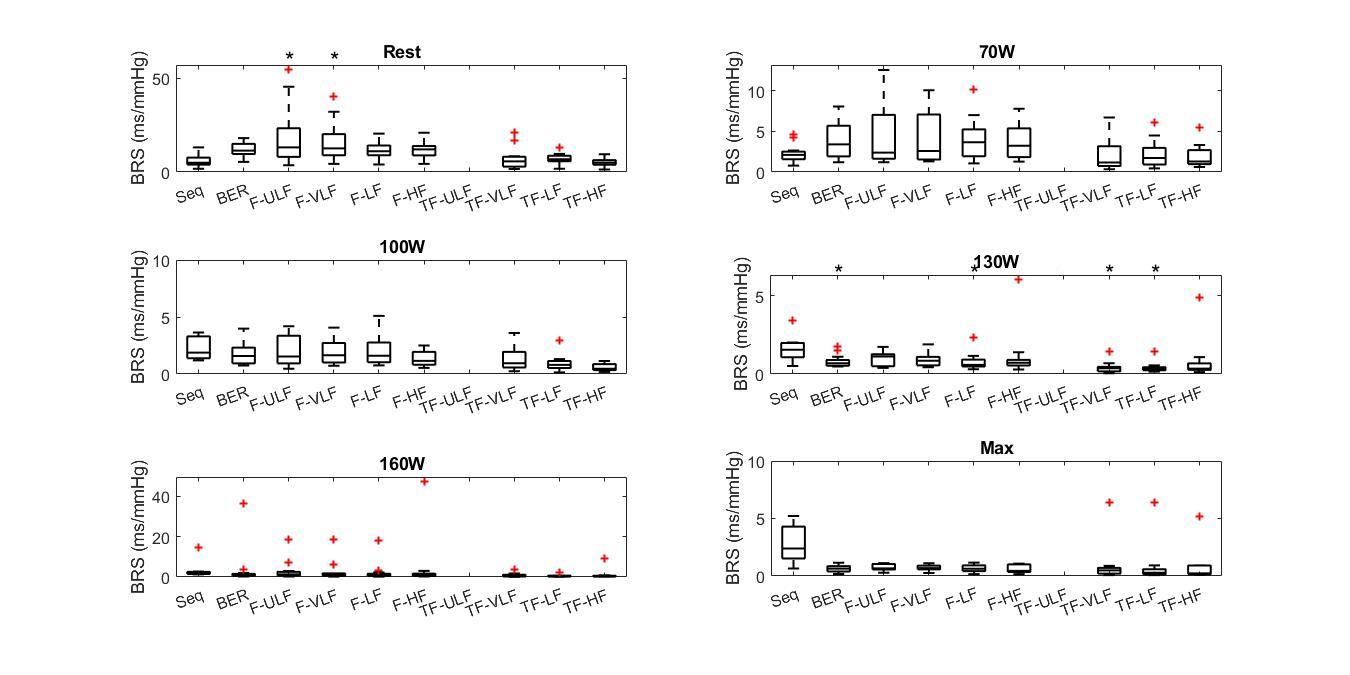

Supplement: FIGURE S4 — Baroreflex sensitivity estimation for the exercise protocol at sea level, respiration removed. +Denotes outliers. ∗Different from Seq (p < 0.05). [file Image_4.JPEG]

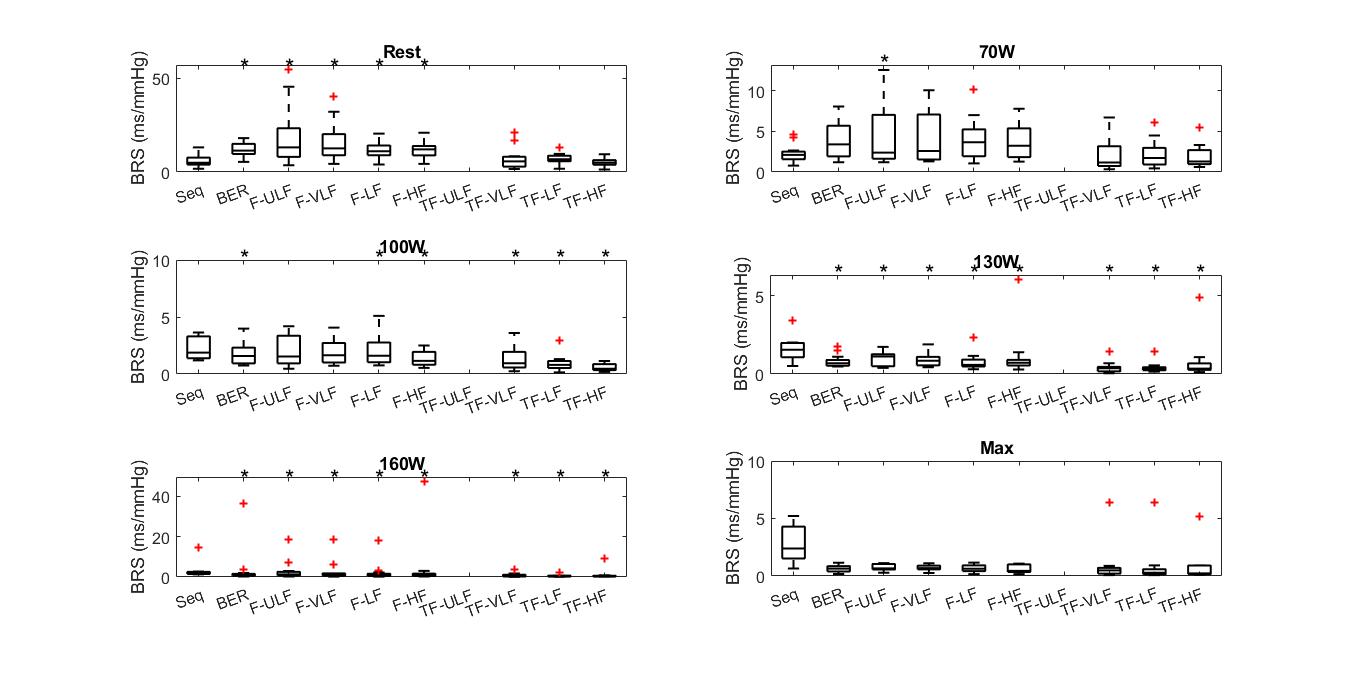

Supplement: FIGURE S5 — Baroreflex sensitivity estimation for the exercise protocol on the first day at 5,260 m, respiration removed. +Denotes outliers. ∗Different from Seq (p < 0.05). [file Image_5.JPEG]

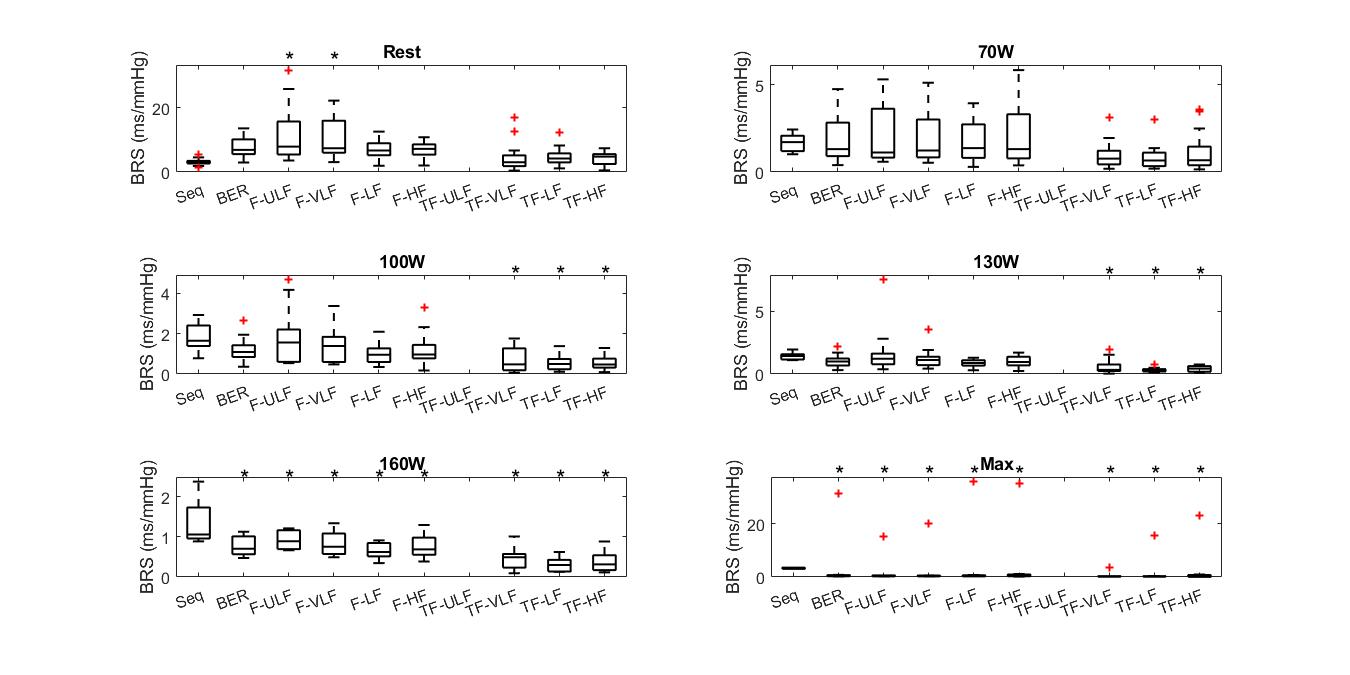

Supplement: FIGURE S6 — Baroreflex sensitivity estimation for the exercise protocol on the 16th day at 5,260 m, respiration removed. +Denotes outliers. ∗Different from Seq (p < 0.05). [file Image_6.JPEG]
