# Supplementary material for: AltitudeOmics: Spontaneous Baroreflex Sensitivity During Acclimatization to 5,260 m: A Comparison of Methods
Source: Front Physiol. 2019 Dec 10;10:1505. doi: 10.3389/fphys.2019.01505 (PMC6914841; doi:10.3389/fphys.2019.01505)
Supplement: TABLE S1 — Bland & Altman parameters during the rest protocol. [file Data_Sheet_1.PDF]

Supplementary Table 1. Bland & Altman parameters during the rest protocol

		**rest**			**clamp-40**			**REB**			**HVE**		
		SL	*ALT1*	*ALT16*	*SL*	*ALT1*	*ALT16*	*SL*	*ALT1*	*ALT16*	*SL*	*ALT1*	*ALT16*
BER	slope	0.98	1.31	0.42	1.45	1.12	0.20	1.02	1.04	1.02	0.93	1.20	0.79
	intercept	1.61	-0.66	4.12	-1.61	0.70	5.89	1.40	1.06	0.97	1.69	0.68	0.45
	R^2	0.54	0.88	0.04	0.66	0.97	0.03	0.67	0.51	0.63	0.94	0.52	0.98
	RMSE	2.11	1.99	1.86	3.19	1.84	3.87	2.69	1.20	1.44	1.62	6.12	1.50
	bias	0.89	0.70	2.27	1.64	1.01	1.26	0.46	1.24	0.93	0.77	0.56	-0.80
	p	0.17	0.51	**<0.01**	0.27	0.25	**0.04**	0.10	**0.04**	0.16	0.33	0.32	0.30
	rpc	4.01	4.48	3.65	6.59	4.14	8.74	5.10	2.27	2.72	3.23	11.74	5.84
	CV	23	33	40	33	17	79	71	38	39	16	72	45
F-ULF	slope	-0.66	1.15	2.57	2.84	0.63	0.28	1.33	0.07	2.00	1.13	1.63	0.52
	intercept	20.02	2.41	3.74	-5.15	11.15	7.45	3.71	7.16	2.71	9.72	-1.53	0.52
	R^2	0.03	0.58	0.04	0.27	0.36	0.06	0.45	<0.01	0.18	0.31	0.41	0.95
	RMSE	9.52	4.12	11.99	14.37	8.46	3.72	5.58	6.12	8.04	12.15	10.26	1.51
	bias	1.47	3.29	6.79	4.81	7.17	3.59	2.78	3.58	2.66	8.31	0.72	-1.59
	p	**0.03**	0.08	**<0.001**	**<0.01**	**0.03**	**<0.001**	**<0.001**	**0.001**	**<0.03**	**<0.01**	0.90	0.07
	rpc	19.47	7.87	22.75	29.31	17.55	8.23	10.86	11.75	15.53	23.12	20.39	12.00
	CV	87	50	136	104	60	63	106	123	134	77	118	107
F-VLF	slope	-0.16	1.24	2.41	2.20	0.62	0.59	1.41	0.63	1.78	0.86	1.46	0.65
	intercept	14.98	2.11	2.92	-3.02	10.30	5.59	2.40	4.06	2.62	10.20	-0.78	0.90
	R^2	<0.01	0.53	0.05	0.36	0.51	0.31	0.61	0.04	0.23	0.31	0.46	0.97
	RMSE	7.41	4.85	9.74	8.97	6.26	2.86	4.25	3.63	6.04	9.35	8.34	1.37
	bias	3.94	2.75	5.16	5.30	4.98	2.97	1.46	2.24	2.75	6.12	0.74	-0.92
	p	**0.03**	0.12	**0.001**	**<0.01**	**0.01**	**<0.01**	**<0.001**	**<0.01**	**0.01**	**<0.01**	0.56	0.39
	rpc	14.94	9.35	18.50	18.43	13.93	5.91	8.59	6.90	11.69	17.85	16.40	8.83
	CV	70	59	121	75	50	47	93	88	108	64	98	71
F-LF	slope	0.85	1.60	0.61	1.50	0.58	0.40	1.35	1.15	0.97	0.93	1.48	0.92
	intercept	4.07	-1.34	4.35	-0.95	6.91	5.16	2.51	1.57	2.02	3.01	-0.24	1.65
	R^2	0.25	0.75	0.06	0.52	0.76	0.09	0.61	0.34	0.56	0.81	0.78	0.98

	RMSE	3.40	3.82	2.15	4.42	3.39	3.99	4.12	1.87	1.58	3.23	4.43	1.59
	bias	2.34	1.43	3.09	2.43	2.47	1.63	1.77	1.80	1.76	2.60	2.34	1.28
	p	**0.02**	0.48	**<0.001**	0.09	0.07	0.06	**0.01**	**0.01**	**0.02**	0.13	0.07	**0.04**
	rpc	6.48	8.61	4.10	8.88	10.28	8.35	8.21	3.56	2.98	6.21	9.66	3.52
	CV	34	58	40	43	42	75	90	53	38	29	56	23
F-HF	slope	1.50	1.00	0.36	1.28	1.53	<0.01	0.86	0.90	0.93	0.88	1.05	1.28
	intercept	-3.15	-0.26	2.16	-1.59	-4.25	5.23	0.98	0.35	0.26	1.61	1.02	-1.09
	R^2	0.75	0.93	0.20	0.80	0.98	<0.01	0.61	0.51	0.69	0.81	0.39	0.99
	RMSE	1.98	0.96	0.68	1.98	2.01	5.20	2.59	1.03	1.14	3.04	6.94	1.66
	bias	0.28	-0.30	-0.07	0.17	0.19	-0.24	-0.12	-0.17	-0.06	-0.05	-0.07	-0.11
	p	0.61	0.97	0.84	0.63	0.97	0.87	0.78	0.74	0.73	0.97	0.97	0.91
	rpc	4.36	1.82	1.68	4.10	10.93	11.49	5.02	1.96	2.17	6.02	13	7.41
	CV	26	15	24	23	46	121	80	40	36	31	84	50
TF-VLF	slope	0.41	0.55	0.59	1.03	<-0.01	0.34	0.83	0.80	0.48	0.58	1.26	0.63
	intercept	4.42	1.87	3.01	-0.97	8.66	2.22	0.78	0.92	1.12	3.08	-4.26	-1.02
	R^2	0.03	0.29	0.02	0.35	<0.01	0.28	0.73	0.14	0.30	0.52	0.50	0.98
	RMSE	5.59	3.64	4.04	4.30	5.89	1.74	1.93	2.32	1.35	3.99	6.68	1.18
	bias	-1.86	-0.71	1.23	-0.85	-2.51	-0.46	0.26	-0.25	-0.27	-0.94	-3.97	-2.68
	p	0.70	0.46	0.21	0.63	0.59	0.56	0.86	0.87	0.63	0.68	**<0.01**	**<0.001**
	rpc	10.93	7.74	7.64	8.15	22	5.16	3.88	4.40	3.13	9.58	12.87	9.20
	CV	70	68	87	49	116	64	65	85	57	53	109	88
TF-LF	slope	0.80	0.98	0.36	0.75	0.31	-0.03	1.01	1.14	0.92	0.65	0.78	0.45
	intercept	2.92	0.36	4.08	3.12	6.46	4.85	0.91	-0.18	0.81	2.65	0.94	2.06
	R^2	0.37	0.66	0.03	0.46	0.50	<0.01	0.77	0.46	0.83	0.79	0.89	0.93
	RMSE	2.41	2.92	1.90	2.51	3.20	2.97	2.09	1.46	0.77	2.45	1.48	1.62
	bias	1.21	-0.20	1.68	1.27	0.14	0.42	0.37	0.15	0.78	-0.32	-0.86	-0.34
	p	0.20	0.93	**0.02**	0.51	0.71	0.60	0.63	0.53	0.39	0.92	0.93	0.94
	rpc	4.66	5.52	3.75	4.97	14.64	8.35	3.97	2.78	1.47	6.69	3.57	13.79
	CV	26	44	42	27	71	92	60	56	23	36	26	113
TF-HF	slope	1.13	0.91	0.36	1.36	1.12	-0.08	0.59	0.71	0.70	0.87	0.90	0.51
	intercept	-1.47	-0.43	1.82	-3.45	-1.78	3.23	0.94	0.21	0.13	0.29	-0.59	0.17

R^2	0.61	0.89	0.16	0.71	0.99	<0.01	0.70	0.72	0.80	0.90	0.72	0.97
RMSE	2.08	1.36	0.75	2.70	1.35	3.36	1.45	0.51	0.65	2.14	3.03	1.24
bias	-0.66	-0.74	-0.70	-0.68	-0.62	-1.28	-0.20	-0.48	-0.54	-0.93	-0.53	-1.35
p	0.70	0.46	0.10	0.58	0.56	**0.04**	0.73	0.09	0.15	0.39	0.26	**0.01**
rpc	3.98	2.68	1.80	5.54	3.39	9.11	4.05	1.16	1.61	4.42	5.79	12.17
CV	25	24	27	33	16	127	74	27	31	24	45	113

slope: slope of the relationship between BRS-Seq and the tested method ; intercept: corresponding y-intercept ; R^2: corresponding squared coefficient of determination ; RMSE: root mean square error ; bias: median bias ; p: p-value for difference between medians ; rpc: reproducibility coefficient (ms/mmHg) ; CV: coefficient of variation (%).

Supplementary table 2. Bland & Altman parameters during the rest protocol, without respiration

		rest			clamp-40			REB			HVE		
		SL	*ALT1*	*ALT16*	*SL*	*ALT1*	*ALT16*	*SL*	*ALT1*	*ALT16*	*SL*	*ALT1*	*ALT16*
BER	slope	-0.52	3.29	1.92	1.43	0.29	1.60	0.83	1.05	0.97	1.86	1.81	2.93
	intercept	13.19	0.75	2.93	7.52	14.19	5.07	2.88	3.16	2.38	4.41	2.07	-2.83
	R^2	0.09	0.22	0.22	0.57	0	0.39	0.22	0.07	0.35	0.28	0.13	0.75
	RMSE	3.69	7.69	2.81	5.46	11.39	12.07	5.83	4.79	2.33	6.90	11.77	5.48
	bias	8.35	5.36	5.05	6.89	7.28	2.67	1.24	1.97	1.82	6.53	2.72	1.43
	p	**<0.01**	**<0.01**	**<0.01**	**<0.01**	**<0.01**	**<0.01**	0.23	**<0.01**	**<0.01**	**<0.01**	**<0.05**	0.06
	rpc	9.55	15.56	5.50	10.95	21.91	23.93	11.14	9.10	4.43	13.62	22.66	15.67
	CV	64	119	60	59	110	168	125	113	62	87	148	152
F-ULF	slope	-1.66	2.49	2.66	2.47	0.86	2.63	0.55	1.29	2.93	3.57	2.23	6.09
	intercept	20.55	4.01	4.48	11.06	14.76	7.45	4.64	4.76	0.46	2.63	0.32	-8.94
	R^2	0.15	0.09	0.09	0.37	0.02	0.59	0.10	0.05	0.43	0.33	0.11	0.68
	RMSE	8.79	9.78	6.62	14.33	14.81	13.31	6.03	7.39	6.07	11.75	16.13	13.32
	bias	8.01	4.38	9.33	12.83	6.26	8.35	1.52	3.33	2.33	9.24	1.77	1.79
	p	**<0.01**	**<0.01**	**<0.01**	**<0.01**	**<0.01**	**<0.01**	0.07	**<0.01**	**<0.01**	**<0.01**	0.12	0.13
	rpc	20.20	18.88	12.81	29.84	28.12	31.39	11.89	14.03	13.24	24.96	31.16	39.89
	CV	115	135	105	112	121	155	122	137	133	122	199	271
F-VLF	slope	-1.15	2.85	2.12	1.94	0.87	1.89	0.82	1.29	1.97	3.17	1.98	5.42
	intercept	17.69	2.86	4.38	8.02	13.35	7.25	3.38	3.76	1.71	3.27	1.20	-7.58
	R^2	0.14	0.12	0.09	0.52	0.04	0.51	0.24	0.06	0.46	0.33	0.11	0.69
	RMSE	6.32	9.36	5.07	8.22	11.52	11.09	5.35	6.42	3.82	10.47	13.93	11.58
	bias	10.18	4.61	7.35	11.71	6.67	7.23	1.53	2.97	2.48	9.79	2.55	1.39
	p	**<0.1**	**<0.01**	**<0.01**	**<0.01**	**<0.01**	**<0.01**	0.10	**<0.01**	**<0.01**	**<0.01**	0.08	0.12
	rpc	15.10	18.29	9.76	17.46	21.87	23.37	10.22	12.20	7.96	22.01	26.83	34.66
	CV	90	131	89	82	100	134	109	132	90	112	175	250
F-LF	slope	-0.29	3.59	1.66	1.02	0.45	1.30	1.36	1.25	0.89	2.21	1.35	3.21
	intercept	12.27	0.27	2.65	7.72	13.43	4.92	1.78	2.99	2.57	3.17	4.57	-3.11
	R^2	0.03	0.27	0.29	0.62	0.01	0.47	0.53	0.06	0.51	0.39	0.10	0.76

	RMSE	3.94	7.38	2.01	3.53	10.69	8.35	4.76	6.33	1.55	6.37	10.08	5.78
	bias	7.76	5.61	3.83	6.81	7.15	4.10	1.77	1.72	2.00	7.29	3.09	2.41
	p	**<0.01**	**<0.01**	**<0.01**	**<0.01**	**<0.01**	**<0.01**	0.10	**<0.01**	**<0.01**	**<0.01**	**0.01**	**0.03**
	rpc	9.28	15.26	3.94	6.70	20.47	16.22	9.37	12.02	2.97	13.20	19.20	17.33
	CV	62	113	47	40	102	124	97	143	42	84	122	159
F-HF	slope	-0.53	3.36	2.02	1.25	0.28	1.68	0.49	1.02	0.60	1.04	1.99	2.45
	intercept	12.41	0.20	1.84	6.19	14.27	4.17	3.41	2.54	2.39	7.12	1.27	-2.02
	R^2	0.04	0.29	0.41	0.66	0.00	0.37	0.10	0.08	0.14	0.10	0.13	0.76
	RMSE	5.84	6.52	1.87	3.94	12.66	13.35	5.60	4.43	2.68	7.38	12.86	4.44
	bias	6.28	4.42	3.83	6.02	7.19	1.93	0.56	1.19	1.02	5.61	2.05	1.08
	p	**<0.01**	**<0.01**	**<0.01**	**<0.01**	**<0.01**	**<0.01**	0.41	**<0.01**	0.07	**<0.01**	0.08	0.20
	rpc	12.86	13.56	3.86	7.78	24.29	26.50	11.21	8.40	5.26	14.01	24.85	12.16
	CV	91	106	46	47	121	194	135	114	85	93	161	129
TF-VLF	slope	-0.58	2.07	0.97	0.49	0.05	1.47	0.55	0.82	0.10	0.27	0.98	4.48
	intercept	9.40	-0.90	2.48	6.82	6.78	3.75	1.54	1.99	2.69	5.16	0.21	-8.14
	R^2	0.06	0.35	0.04	0.14	0.00	0.41	0.34	0.03	0.02	0.02	0.09	0.65
	RMSE	5.31	3.53	3.53	5.27	4.51	10.65	2.89	5.48	1.45	4.91	7.74	10.52
	Bias	1.17	1.02	2.27	4.77	1.46	1.22	-0.03	0.03	1.07	1.50	-0.85	-1.14
	p	0.24	**0.02**	**0.04**	**<0.01**	0.13	**<0.01**	0.94	0.65	0.17	0.26	0.09	0.41
	rpc	12.13	7.15	6.71	10.87	9.79	20.91	6.31	10.40	4.10	9.84	14.69	29.05
	CV	111	90	101	81	84	168	94	164	77	98	153	285
TF-LF	slope	-0.45	2.22	1.47	0.26	0.44	0.76	0.92	0.68	0.49	0.05	0.82	1.71
	intercept	10.47	-0.09	1.40	8.19	6.95	3.12	0.56	2.12	1.98	6.95	2.13	-1.39
	R^2	0.08	0.44	0.29	0.10	0.04	0.44	0.50	0.02	0.31	0.00	0.08	0.76
	RMSE	3.43	3.12	1.79	3.43	5.68	5.25	3.41	6.01	1.32	3.42	6.70	3.07
	bias	5.39	2.86	2.05	4.40	2.45	0.72	0.49	-0.15	0.01	4.08	0.30	0.45
	p	**<0.01**	**0.01**	**<0.01**	**<0.01**	**0.01**	0.09	0.99	0.61	0.08	**<0.01**	0.73	0.86
	rpc	9.01	6.58	3.47	8.97	11.14	10.31	6.49	11.43	3.04	7.70	12.74	7.24
	CV	72	71	52	65	82	110	93	186	54	70	119	95
TF-HF	slope	-0.43	2.09	1.23	0.59	0.92	1.05	0.26	0.72	0.44	0.75	1.01	0.78
	intercept	9.31	0.86	1.46	5.24	5.59	2.44	1.99	1.33	1.17	3.82	1.70	0.28

R^2	0.03	0.28	0.41	0.45	0.12	0.32	0.09	0.05	0.29	0.19	0.09	0.70
RMSE	5.85	4.12	1.14	2.85	6.46	9.21	3.04	4.18	1.22	3.59	8.03	1.61
bias	2.74	2.78	1.91	2.63	3.69	0.04	-0.81	-0.33	-0.27	2.51	0.33	-0.68
p	**0.01**	**<0.01**	**<0.01**	**<0.01**	**<0.01**	0.73	0.84	0.33	0.92	**0.01**	0.78	0.47
rpc	12.68	8.22	2.19	6.41	12.26	17.48	7.75	7.96	2.99	6.90	15.24	3.34
CV	111	84	35	52	84	180	125	146	64	65	136	54

slope: slope of the relationship between BRS-Seq and the tested method ; intercept: corresponding y-intercept ; R^2: corresponding squared coefficient of determination ; RMSE: root mean square error ; bias: median bias ; p: p-value for difference between medians ; rpc: reproducibility coefficient (ms/mmHg) ; CV: coefficient of variation (%).

Supplementary table 3. Bland & Altman parameters during the exercise protocol

		**rest**			**70 W**			**100 W**			**130 W**			**160 W**			**Max**		
		SL	*ALT1*	*ALT16*	*SL*	*ALT1*	*ALT16*	*SL*	*ALT1*	*ALT16*	*SL*	*ALT1*	*ALT16*	*SL*	*ALT1*	*ALT16*	*SL*	*ALT1*	*ALT16*
BER	slope	0.35	0.14	0.88	0.06	-0.08	0.69	0.56	0.43	-0.04	0.03	0.55	-0.58	0.15	2.79	0.03	>10	0.27	0.09
	intercept	7.73	5.98	2.90	2.79	1.65	0.83	0.42	0.44	1.45	0.65	0.10	1.81	0.61	-1.83	0.76	>10	0.16	0.23
	R^2	0.16	0.45	0.56	0.01	0.01	0.61	0.32	0.70	0.03	0.05	0.98	0.55	0.67	0.35	0.01	0.02	0.99	0.13
	RMSE	3.48	0.82	1.66	1.50	0.77	0.88	0.67	0.18	0.18	0.22	0.03	0.36	0.45	0.23	0.16	>100	0.01	0.07
	bias	1.47	-0.46	2.52	-0.40	-0.10	-0.22	-0.32	-0.26	-1.09	-1.02	-0.40	-0.33	-0.44	-0.31	-0.47	-0.49	-0.85	-0.45
	p	0.51	0.99	0.42	0.96	0.99	0.69	0.19	0.99	0.10	**<0.05**	0.20	0.31	0.16	0.10	0.10	0.13	0.10	**<0.05**
	rpc	8.14	6.35	2.86	4.27	1.68	1.71	1.39	0.56	1.48	3.07	0.21	1.96	6.57	0.35	1.12	>100	0.50	0.50
	CV	38	44	20	71	56	24	38	30	40	110	13	83	197	26	52	>500	30	46
F-ULF	slope	-0.19	-0.59	0.74	<0.01	-2.18	0.93	0.46	-0.14	-2.89	-0.21	1.55	-1.05	-0.06	-1.15	0.21	>10	-1.62	0.17
	intercept	19.78	13.36	6.38	3.15	8.27	-0.05	0.90	2.06	9.57	1.53	0.10	3.04	1.84	2.16	0.94	>10	2.93	0.35
	R^2	<0.01	0.63	0.06	<0.01	0.58	0.28	0.11	0.09	0.65	0.37	0.58	0.57	0.06	0.16	0.18	0.02	0.96	0.04
	RMSE	13.59	2.40	6.13	2.33	1.61	2.39	1.08	0.28	1.72	0.48	0.43	0.63	1.01	0.16	0.30	>100	0.17	0.25
	bias	4.94	0.73	5.29	-0.01	3.56	-0.39	-0.52	1.17	0.03	-0.87	0.54	0.30	0.08	0.38	-0.36	0.05	-0.81	-0.27
	p	0.28	0.40	0.22	0.99	0.10	0.42	0.51	0.10	0.99	0.10	0.10	0.69	0.44	0.10	0.69	0.72	0.70	0.15
	rpc	26.42	12.06	10.45	5.49	4.41	4.06	2.12	1.08	6.15	3.90	0.65	2.64	8.33	0.29	1.01	>100	1.82	0.61
	CV	95	74	62	89	70	57	54	38	124	124	25	91	206	14	39	>500	86	48
F-VLF	slope	-0.18	-0.24	0.59	0.01	-0.04	0.85	0.48	0.17	-1.71	-0.12	0.44	-0.84	0.04	4.96	0.04	>10	-0.02	0.07
	intercept	17.21	10.85	5.98	2.81	2.67	0.41	0.72	0.88	5.82	1.09	0.31	2.40	0.91	-3.46	0.76	>10	0.49	0.26
	R^2	0.01	0.19	0.10	<0.01	0.01	0.56	0.18	0.07	0.71	0.32	0.50	0.54	0.12	0.97	0.03	0.02	0.11	0.05
	RMSE	9.75	2.62	3.79	1.85	0.36	1.20	0.85	0.40	0.90	0.30	0.14	0.54	0.48	0.05	0.17	>100	0.03	0.09
	bias	4.82	0.38	3.47	-0.44	1.14	-0.19	-0.45	0.21	-1.06	-0.99	-0.20	-0.06	-0.21	-0.08	-0.50	-0.41	-0.94	-0.36
	p	0.28	0.70	0.15	0.80	0.10	0.84	0.33	0.99	0.31	**0.03**	0.20	0.84	0.57	0.70	0.10	0.19	0.10	0.01
	rpc	20.00	9.77	6.61	4.81	1.34	2.07	1.73	0.92	4.06	3.56	0.32	2.35	7.40	0.34	1.11	>100	0.71	0.52
	CV	79	60	43	81	33	29	46	46	100	122	19	91	219	22	51	>500	42	47
F-LF	slope	0.21	0.42	0.57	0.02	0.36	0.64	0.59	0.28	0.20	-0.03	0.20	-0.50	0.18	3.58	-0.02	>10	0.17	0.04
	intercept	9.37	4.77	5.10	2.64	0.24	1.00	0.37	0.19	0.69	0.67	0.11	1.45	0.32	-2.61	0.51	>10	0.01	0.17
	R^2	0.11	0.66	0.26	<0.01	0.72	0.34	0.38	0.67	0.10	0.02	0.24	0.65	0.78	0.73	0.01	0.02	0.99	0.11
	RMSE	2.71	1.60	2.05	1.27	0.19	1.41	0.62	0.13	0.48	0.30	0.12	0.26	0.41	0.13	0.16	>100	0.01	0.04
	bias	1.76	-0.42	2.00	-0.44	-0.62	-0.92	-0.15	-0.61	-1.40	-0.98	-0.69	-0.47	-0.60	-0.50	-0.76	-0.63	-1.13	-0.55
	p	0.38	0.99	0.06	0.88	0.10	0.84	0.28	0.20	0.03	**<0.05**	0.10	0.06	0.05	0.10	**<0.05**	0.13	0.10	**<0.05**
	rpc	7.94	4.78	3.80	4.11	0.81	2.59	1.29	0.66	1.38	3.27	0.40	1.82	6.32	0.28	1.17	>100	0.57	0.51
	CV	36	31	26	71	36	37	35	45	40	121	31	87	202	23	64	>500	40	52
F-HF	slope	1.05	0.25	0.94	0.08	0.88	1.18	0.62	0.12	0.98	0.20	0.18	-0.02	0.25	7.33	-0.18	>10	0.74	0.07
	intercept	-1.62	2.96	0.48	2.78	-0.18	-0.24	0.06	0.50	-1.00	0.33	0.20	0.73	0.32	-5.74	0.82	>10	-0.44	0.20
	R^2	0.83	0.86	0.96	0.01	0.92	0.46	0.35	0.07	0.47	0.44	0.36	<0.0	0.82	0.78	0.11	0.02	0.95	0.07
	RMSE	2.05	0.53	0.43	1.61	0.22	2.00	0.69	0.28	0.85	0.39	0.08	0.37	0.50	0.24	0.34	>100	0.09	0.08
	bias	-1.50	-1.72	0.13	-0.38	-0.47	0.78	-0.67	-0.14	-1.13	-0.90	-0.69	-0.46	-0.58	-0.60	-0.87	-0.70	-0.76	-0.44
	p	0.51	0.40	0.99	0.88	0.40	0.42	0.05	0.20	0.10	**<0.05**	0.10	0.06	0.05	0.20	**<0.05**	0.28	0.10	**<0.05**

	rpc	3.73	5.55	0.76	4.33	0.34	3.43	1.39	0.87	1.45	2.61	0.39	1.36	5.84	0.63	1.43	>100	0.21	0.51
	CV	19	43	6.19	71	13	44	41	54	40	92	29	66	178	51	74	>500	13	49
TF-VLF	slope	-0.08	-0.06	-0.23	0.03	-0.30	0.40	0.07	0.43	-0.33	0.02	1.52	-0.62	0.01	-4.15	0.53	>10	-0.64	0.20
	intercept	8.35	3.85	5.70	1.42	2.03	-0.20	0.86	0.38	1.31	0.34	-1.07	1.57	0.49	3.83	-0.20	>10	1.00	-0.02
	R^2	<0.01	0.10	0.09	<0.01	0.10	0.73	0.01	0.60	0.68	0.02	0.54	0.46	0.03	0.78	0.87	0.02	0.99	0.38
	RMSE	7.40	1.04	1.54	1.74	0.80	0.39	0.69	0.22	0.19	0.20	0.46	0.47	0.34	0.13	0.14	>100	0.01	0.08
	Bias	-4.68	-2.51	-1.45	-1.79	0.01	-2.83	-1.01	-0.36	-1.95	-1.48	-0.65	-0.74	-0.63	-0.61	-0.83	-0.55	-1.26	-0.62
	p	0.08	0.10	0.22	**<0.05**	0.70	**<0.05**	**<0.05**	0.99	**<0.01**	**<0.01**	0.20	0.06	**<0.05**	0.10	**<0.05**	0.13	0.10	**<0.01**
	rpc	16	7.89	5.13	4.62	1.91	1.74	1.88	0.59	1.88	3.10	0.68	2.07	7.59	0.47	0.57	>100	1.13	0.44
	CV	90	72	50	100	63	35	62	33	66	126	49	101	262	41	30	>500	79	48
TF-LF	slope	0.16	0.03	0.76	-0.19	0.17	0.21	0.05	0.27	-0.16	0.04	0.24	-0.17	0.05	-0.91	0.04	>10	-0.06	**<0.01**
	intercept	6.99	5.09	2.59	2.22	0.26	1.01	0.60	0.08	1.00	0.27	-0.07	0.61	0.17	0.87	0.21	>10	0.14	0.08
	R^2	0.06	0.84	0.35	0.15	0.66	0.37	0.01	0.49	0.14	0.23	0.71	0.69	0.55	0.78	0.08	0.02	0.72	**<0.01**
	RMSE	2.78	0.07	2.18	0.88	0.10	0.42	0.44	0.18	0.33	0.11	0.05	0.08	0.17	0.03	0.09	>100	0.02	0.03
	bias	-1.07	-1.50	1.06	-1.13	-0.76	-1.79	-1.31	-0.77	-2.24	-1.26	-0.87	-0.76	-0.85	-0.72	-1.07	-0.87	-1.34	-0.65
	p	0.38	0.70	0.42	**<0.05**	0.10	0.10	**<0.01**	0.10	**<0.05**	**<0.01**	0.10	**<0.05**	**<0.01**	0.10	**<0.05**	0.08	0.10	0.01
	rpc	8.33	7.07	3.79	4.46	1.01	2.25	1.64	0.69	1.71	3.03	0.35	1.38	7.33	0.17	1.08	>100	0.73	0.53
	CV	44	55	29	95	50	41	59	51	58	125	31	80	276	18	66	>500	58	62
TF-HF	slope	0.10	0.25	0.43	0.37	0.62	0.81	0.23	0.01	0.71	0.10	0.10	0.21	0.15	0.09	0.05	>10	0.11	0.10
	intercept	4.99	1.71	1.70	0.71	-0.30	-0.79	0.20	0.39	-0.91	0.23	0.14	0.14	0.24	0.05	0.21	>100	0.04	0.06
	R^2	0.05	0.94	0.79	0.45	0.96	0.42	0.38	<0.01	0.36	0.26	0.03	0.21	0.62	0.38	0.01	0.02	0.20	0.64
	RMSE	2.01	0.33	0.47	0.78	0.11	1.52	0.24	0.29	0.77	0.28	0.18	0.29	0.50	0.01	0.30	>100	0.11	0.02
	bias	-4.34	-3.10	-1.66	-0.87	-0.80	-1.75	-1.21	-0.33	-1.85	-1.27	-0.79	-0.69	-0.83	-0.71	-1.07	-0.86	-1.22	-0.60
	p	**<0.05**	0.10	0.22	0.13	0.10	0.22	**<0.01**	0.20	**<0.05**	**<0.01**	0.10	**<0.05**	**<0.05**	0.10	**<0.05**	0.08	0.10	**<0.05**
	rpc	7.97	5.50	2.18	2.61	0.48	2.62	1.24	0.96	1.37	2.88	0.48	1.05	6.60	0.08	1.17	>100	0.63	0.48
	CV	49	49	21	53	22	44	46	69	45	114	40	59	222	8	71	>500	46	52

slope: slope of the relationship between BRS-Seq and the tested method ; intercept: corresponding y-intercept ; R^2: corresponding squared coefficient of determination ; RMSE: root mean square error ; bias: median bias ; p: p-value for difference between medians ; rpc: reproducibility coefficient (ms/mmHg) ; CV: coefficient of variation (%).
